# Targeting the ectopy‐triggering ganglionated plexuses without pulmonary vein isolation prevents atrial fibrillation

**DOI:** 10.1111/jce.14870

**Published:** 2021-01-19

**Authors:** Belinda Sandler, Min‐Young Kim, Markus B. Sikkel, Louisa Malcolme‐Lawes, Michael Koa‐Wing, Zachary I. Whinnett, Clare Coyle, Nick W. F. Linton, Phang B. Lim, Prapa Kanagaratnam, other members of the Imperial College London, Cardiovascular Study Group/Consortium

**Affiliations:** ^1^ Myocardial Function Section, National Heart and Lung Institute Imperial College London London UK; ^2^ Imperial Centre for Cardiac Engineering Imperial College London London UK; ^3^ Department of Cardiology, Hammersmith Hospital Imperial College Healthcare NHS Trust London UK

**Keywords:** atrial fibrillation, autonomic nervous system, catheter ablation, ganglionated plexus, pulmonary vein ectopy

## Abstract

**Background:**

Ganglionated plexuses (GPs) are implicated in atrial fibrillation (AF). Endocardial high‐frequency stimulation (HFS) delivered within the local atrial refractory period can trigger ectopy and AF from specific GP sites (ET‐GP). The aim of this study was to understand the role of ET‐GP ablation in the treatment of AF.

**Methods:**

Patients with paroxysmal AF indicated for ablation were recruited. HFS mapping was performed globally around the left atrium to identify ET‐GP. ET‐GP was defined as atrial ectopy or atrial arrhythmia triggered by HFS. All ET‐GP were ablated, and PVs were left electrically connected. Outcomes were compared with a control group receiving pulmonary vein isolation (PVI). Patients were followed‐up for 12 months with multiple 48‐h Holter ECGs. Primary endpoint was ≥30 s AF/atrial tachycardia in ECGs.

**Results:**

In total, 67 patients were recruited and randomized to ET‐GP ablation (*n* = 39) or PVI (*n* = 28). In the ET‐GP ablation group, 103 ± 28 HFS sites were tested per patient, identifying 21 ± 10 (20%) GPs. ET‐GP ablation used 23.3 ± 4.1 kWs total radiofrequency (RF) energy per patient, compared with 55.7 ± 22.7 kWs in PVI (*p* = <.0001). Duration of procedure was 3.7 ± 1.0 and 3.3 ± 0.7 h in ET‐GP ablation group and PVI, respectively (*p* = .07). Follow‐up at 12 months showed that 61% and 49% were free from ≥30 s of AF/AT with PVI and ET‐GP ablation respectively (log‐rank *p* = .27).

**Conclusions:**

It is feasible to perform detailed global functional mapping with HFS and ablate ET‐GP to prevent AF. This provides direct evidence that ET‐GPs are part of the AF mechanism. The lower RF requirement implies that ET‐GP targets the AF pathway more specifically.

## 1 INTRODUCTION

Atrial fibrillation (AF) is the most common type of arrhythmia that causes stroke and discomfort in patients and poses a significant health burden around the world.^1^ Pulmonary vein (PV) ectopy is the most common trigger for AF,^2^ and complete pulmonary vein isolation (PVI) has been the standard treatment for drug‐refractory AF for almost two decades. Clinical trials have repeatedly shown that PVI improves symptoms and lessens the burden of AF.^3^ However, 40%–50% of patients after their first procedure have a recurrence of AF,^4^, ^5^ and patients with or without AF frequently have electrically re‐connected PVs.^6^, ^7^ This suggests that the success of AF ablation is more complex than a simple PVI‐based theory.

The ganglionated plexuses (GPs) which are part of the intrinsic cardiac autonomic nervous system have often been cited as being an important component of AF initiation and maintenance theories.^8^ GPs comprise dense sympathetic and parasympathetic nerves and are situated in the epicardial fat pad of human hearts.^9^ GPs are interconnected with one another^10^ and nerves communicating with GPs penetrate through all levels of the atria. This allows for stimulation from the endocardium, which leads to the release of acetylcholine and catecholamines that shorten the atrial refractory period and causes PV ectopy and AF.^11^

There are different stimulation techniques to localize GPs. The most common method involves delivering high‐frequency stimulation (HFS; 10–14 V, 20 Hz) continuously for up to 10 s (continuous HFS), or until >50% of RR prolongation is observed from the baseline with atrioventricular dissociation.^12^ This causes asystole for up to several seconds with a blood pressure drop. We previously mapped atrioventricular dissociating GP (AVD‐GP) in the human left atrium and found that they occupy discrete anatomical regions, and do not conform to all the “common GP cluster” regions as described from previous topographical studies.^13^

Another method of localizing GPs is using HFS within the local refractory period.^14^ This technique paces the atrium at a fixed rate, and 100‐ms duration of HFS is delivered at 10–14 V, 20 Hz (synchronized HFS). This avoids direct myocardial capture and has been shown to trigger PV and non‐PV ectopy that can lead to AF.^15^ We previously mapped these ectopy‐triggering GPs (ET‐GP) which were anatomically and functionally distinct from AVD‐GP.^16^ ET‐GP have not been widely studied in patients even though the effects are comparable to clinical episodes of AF.

To test the hypothesis that ET‐GP are part of the triggering mechanism for AF, we performed selective endocardial ablation of ET‐GP and monitored AF recurrences.

## 2 METHODS

This was a prospective, single‐center study recruiting patients with paroxysmal AF indicated for AF ablation. All patients gave written informed consent. The study was approved by the Local Research Ethics Committee and the Health Research Authority. This was a pilot study for the clinical trial registered on ClinicalTrials.gov (NCT02487654). The study procedures took place between December 2013 and May 2017.

Patients were randomized to either ET‐GP ablation without PVI or to a control arm of standard PVI. The purpose of the control arm was to assess the ethical justification of the study, and an independent Data and Safety Monitoring Board (two electrophysiologists, one general cardiologist) reviewed outcomes after recruiting 20 consecutive patients. This study was not powered for sample size, as it is a proof‐of‐concept study to first establish the safety and feasibility of ET‐GP ablation, and generate outcome data for future powered studies.

We performed block randomization using the “sealed envelope” approach. Patients and their cardiologists providing their usual care were blinded to their randomization. The inclusion and exclusion criteria are included in Table S1. All patients stopped their antiarrhythmics for at least 48 h before their procedures.

All had general anesthesia and transoesophageal echocardiogram (TOE) to rule out left atrial appendage thrombus at the start of their procedures. Transseptal punctures were guided by TOE and fluoroscopy to access the left atrium. We used the CARTO™ system (Biosense Webster Inc.) for 3D electroanatomical mapping of the left atrium. Intracardiac electrograms were recorded at 1000 Hz by the electrophysiology recording system (Bard EP).

### 2.1 ET‐GP mapping with synchronized HFS

Patients randomized to ET‐GP ablation were required to be in sinus rhythm, to pace their atrium. If in AF at the start of the protocol, patients were electrically cardioverted to restore sinus rhythm. A 20‐pole circumferential catheter (LassoNav; Biosense Webster Inc.) was inserted into the nearest PV to where HFS was being tested. This was to maximize the chances of identifying the earliest triggered PV ectopy with HFS, with the assumption that GPs are more likely to have neural connections to adjacent structures.

**Figure 1:**
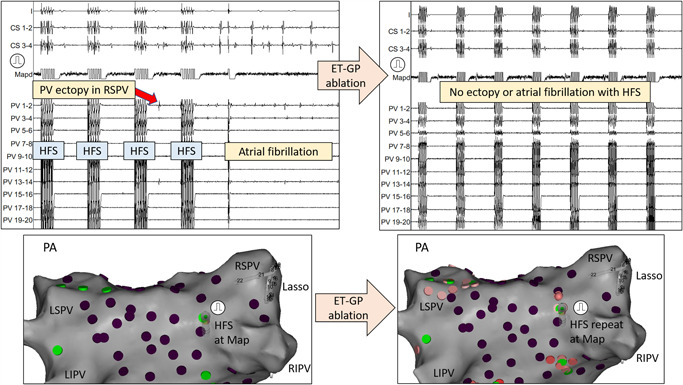
An example of mapping for an ET‐GP site using synchronized HFS is shown on the left‐hand side. PA view of the left atrium at the bottom shows that the ablation catheter (Map) was positioned in the RIPV roof. The pulmonary vein catheter (Lasso) was inserted into the RSPV. Pacing was performed first, followed by delivery of HFS coupled to each pacing stimulus (synchronized HFS). After the third HFS train, PV ectopy was initiated (earliest PV 13–14) which triggered AF. This site was marked as an “ET‐GP” site, tagged green in the CARTO™ geometry. After performing ablation at this site, retesting with synchronized HFS could not trigger the same response as before ablation. This confirmed adequate ablation at this site. We mapped and ablated the rest of the ET‐GPs this way. Purple tags on CARTO represented negative responses to HFS. ET‐GP, ectopy‐triggering ganglionated plexus, HFS, high‐frequency stimulation; PA, posterior–anterior; PV, pulmonary vein; RIPV, right inferior pulmonary vein; RSPV, right superior pulmonary vein

Pacing was performed from the ablation catheter (bipolar 3.5‐mm irrigated tip contact force‐sensing ablation catheter; Smart‐Touch; Biosense Webster Inc.), at a rate higher than the intrinsic rate. A minimum contact force of 3*g* was required before delivering HFS. The Grass S88 stimulator (Astro‐Med) was used to deliver HFS from each pacing stimulus (“synchronized” to pacing, delivering HFS within the local atrial refractory period), for 100–120 ms duration at 40 Hz, 10 V, up to 15 trains^17^ (synchronized HFS). There remained a risk of local myocardial capture if there was enough shortening of the local refractory period. These could be identified by the mapping catheter having the earliest signal with no delay from the last pacing artifact. If this occurred, we repeated HFS and reduced the duration of HFS until no further local capture was evident. A “positive” response to synchronized HFS included single atrial ectopy, few beats of atrial ectopy, atrial tachycardia or AF.^15^ These positive responses were tagged as “ET‐GP” sites on the CARTO geometry. An example of this is shown in Figure 1 (left panel). If there was <3 atrial ectopy triggered with HFS, we retested with HFS up to three times to exclude the possibility of this being due to spontaneous ectopy or mechanical irritation.

Sometimes, patients developed sustained AF that would not self‐terminate within a few minutes of waiting. If this occurred, DC cardioversion was undertaken to restore sinus rhythm, and mapping resumed with synchronized HFS again.

### 2.2 AVD‐GP mapping with continuous HFS

We included all “learning curve” procedures in the ET‐GP ablation group for the per‐protocol analysis. The main procedural hurdle was obtaining limited ET‐GP maps due to sustained AF despite multiple cardioversions. Initially, these patients were crossed over to PVI if they had already undergone three DC cardioversions for sustained AF or had incessant AF that could not be cardioverted. However, we previously showed that a proportion of ET‐GP co‐locates with AVD‐GP.^16^ Therefore, a protocol amendment was instituted, and patients randomized to ET‐GP ablation with sustained AF had AVD‐GP mapping instead of crossing over to PVI. Randomization was also later modified to 2:1 in favor of ET‐GP to offset these early crossovers.

**Figure 2:**
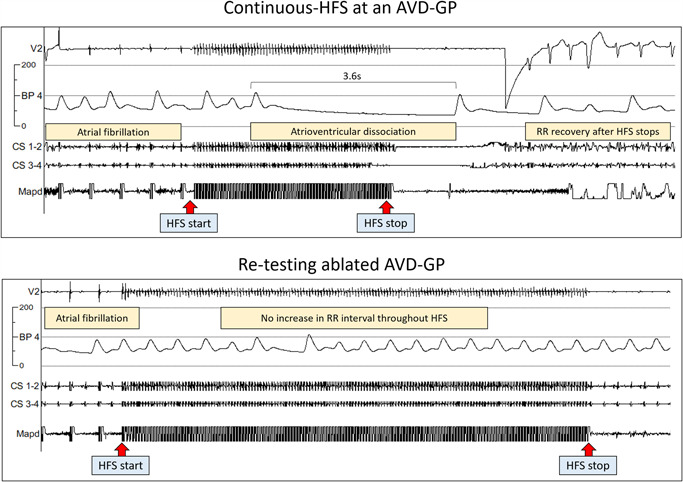
Mapping for AVD‐GP and testing after ablation. During AF, continuous HFS was performed to identify AVD‐GPs as in the top panel. Here, we paced five times to ensure that there was no ventricular capture. A continuous train of HFS was then delivered at the distal poles of an ablation catheter (Mapd). A significant AV dissociation occurred, causing asystole of 3.6 s. We stopped HFS at this point and there was a rapid RR interval recovery and continuation of AF. This site was determined as an AVD‐GP site and ablated at the end of the procedure. The bottom panel shows that retesting with continuous HFS at this ablated site did not trigger any AV dissociation again. This confirmed adequate ablation at this site. We mapped and ablated the rest of AVD‐GPs this way. AVD‐GP, atrioventricular dissociating ganglionated plexus; BP, blood pressure; HFS, high‐frequency stimulation

To map AVD‐GP, a quadripolar catheter was inserted into the right ventricular apex or a continuous arterial blood pressure monitoring was visualized alongside the intracardiac electrograms. This allowed for a more accurate assessment of any significant RR prolongation through the HFS artifact noise. With a minimum 3*g* contact force at the tip of the ablation catheter, HFS was delivered at 40 Hz, 10 V, up to 10 s continuously (continuous HFS). A “positive” response to continuous HFS was defined as a site causing >50% increase in the average RR interval during HFS when compared with 10 RR intervals before HFS.^13^ These were marked as “AVD‐GP” on the CARTO 3D geometry. An example of this is shown in Figure 2 (top panel).

### 2.3 Ablation of GP

A target of at least 80 evenly distributed sites were tested around the left atrium. After completing mapping, both ET‐GP and AVD‐GP were ablated. Clusters of ablation lesion were delivered at each GP site, which involved 30‐s radiofrequency (RF) ablation at each lesion for a minimum of three lesions per GP, and with minimal contact force >3*g*. Posterior wall GPs were ablated with power limited to 25 W. After completing ablation, all sites were retested for a positive response with either synchronized HFS (Figure 1; right panel) or continuous HFS (Figure 2; bottom panel) according to the pre‐ablation characterization.

If sites still triggered ectopy/AT/AF, then further ablation was performed until no further response to HFS was evident. PVs were checked to confirm all remained connected.

### 2.4 Pulmonary vein isolation

Patients randomized to PVI had circumferential ablation around the antra of the PVs with RF energy. A 3.5‐mm irrigated tip contact force‐sensing ablation catheters (Smart‐Touch) with 10–20*g* contact force and 17 ml/min flow was used to perform circumferential antral ablation. Entry block of the PVs confirmed complete PVI using a circular PV mapping catheter.

### 2.5 Clinical follow‐up

All patients were followed‐up every 3 months postablation with 48‐h Holter monitoring and telephone consultations up to 12 months. Patients were encouraged to have additional investigations if symptoms reported between 3 monthly follow‐ups. Any arrhythmia within the first 3 months of “blanking period” was discounted as an endpoint. All patients for repeat ablations received PVI without additional GP ablation.

### 2.6 Definition of endpoint

The primary end‐point was defined as any ≥30 s of AF or AT documented on electrocardiography, Holters, or on any implantable devices such as pacemakers or loop recorders^1^ and/or referral for repeat AF/AT ablations on symptomatic grounds. The secondary endpoint was complications such as significant groin hematoma, pericardial effusion or cardiac tamponade requiring drainage, stroke, myocardial infarction, esophageal injury or fistula, and death.

### 2.7 Statistical analysis

Statistical analysis was performed using GraphPad Prism 5 (GraphPad Inc.). Continuous variables were expressed as mean ± *SD*. Categorical variables were expressed as numbers and percentages. Mann–Whitney *U* test, Fisher's exact test, and unpaired *t* test were used for comparison of means. The primary end‐point analysis was conducted using the intention‐to‐treat (ITT) and per‐protocol (PP) study populations. ITT analysis included patients who were crossed over from their originally randomized group, and PP analysis excluded all crossed over patients. The primary endpoint for ITT and PP study populations were each plotted onto a Kaplan–Meier curve to estimate the event‐free survival rate in each group. *p* < .05 indicated statistical significance.

## 3 RESULTS

**Figure 3:**
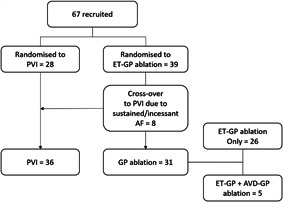
Study flowchart. We recruited 67 patients, of which 8 initially randomized to ET‐GP were crossed over to the PVI group due to sustained AF precluding completion of ET‐GP mapping protocol. After changing our protocol to allow completion of GP mapping in AF, we randomized patients 2:1 for GP ablation until more equal distribution of patients into each group. The final number of patients receiving PVI and GP ablation were 36 and 31, respectively. Five patients from the GP ablation group had a combination of ET‐GP and AVD‐GP ablation. AF, atrial fibrillation; AVD‐GP, atrioventricular‐dissociating ganglionated plexus; ET‐GP, ectopy‐triggering ganglionated plexus; GP, ganglionated plexus; PVI, pulmonary vein isolation

In total, 67 patients were recruited to the study and randomized to ET‐GP ablation (*n* = 39) or PVI (*n* = 28). Initially, eight patients randomized to ET‐GP ablation were crossed over to PVI due to sustained AF despite multiple cardioversions, preventing completion of the ET‐GP protocol (Figure 3). Subsequently, a new protocol was implemented to prevent further cross‐overs due to sustained AF by including AVD‐GP mapping and ablation, as described in Section Methods, 2. Patients were 60 ± 11 years and 63% were male. BMI was 28.6 ± 4.6 kg/m^2^, left atrial diameter size: 3.8 ± 0.4 mm, left ventricular systolic function: 64.1 ± 2.7%, and CHA_2_DS_2_‐VASc score: 1.3 ± 1.1. There were no significant differences in the baseline characteristics between the two groups (Table 1). Most ET‐GP were present around the PV antra, except for the posterior antrum of the right inferior PV. Further ET‐GP were clustered across the roof and in the mid‐anterior wall.

**Table 1:** Demographics of patients

Demographics	All (*n* = 67)	PVI (*n* = 36)	GP ablation (*n* = 31)	*p* value
Age (years)	60 ± 10.8	61.7 ± 11.0	58.1 ± 10.5	.16
Male	42 (63)	20 (56)	22 (71)	.22
BMI (kg/m^2^)	28.6 ± 4.6	29.4 ± 5.2	28 ± 3.8	.34
LA diameter (mm)	3.8 ± 0.4	3.9 ± 0.4	3.7 ± 0.4	.11
LVEF (%)	64.1 ± 2.7	63.8 ± 3.5	64.6 ± 1.2	.34
CHA_2_DS_2_‐VASc	1.3 ± 1.1	1.5	1.1	.21
HTN	27 (40)	15 (42)	12 (39)	1.00
IHD	8 (12)	6 (17)	2 (6)	.27

*Note*: Values are presented as mean ± *SD* or *n* (%).

Abbreviations: BMI, body mass index; CHA_2_DS_2_‐VASc, congestive heart failure, hypertension, age ≥ 74 years, diabetes mellitus, prior stroke, transient ischemic attack, or thromboembolism, vascular disease, age: 65–74 years, sex female; GP, ganglionated plexus; HTN, hypertension; IHD, ischemic heart disease; LA, left atrial; LVEF, left ventricular ejection fraction; RF, radiofrequency.

### 3.1 Ablation procedures and complications

There were 26 patients who had ET‐GP ablation and five patients who had a combination of ET‐GP and AVD‐GP ablations. We tested total of 2787 HFS sites, which identified 570 (20%) GPs. In total, 502 were ET‐GPs and 68 were AVD‐GPs. Patients had on average 103 ± 28 HFS sites tested which identified 21 ± 10 (20%) GPs per patient. Retesting with HFS at all ablated GP sites confirmed no further inducible atrial arrhythmia.

On a PP basis, the duration of the procedure in the ET‐GP and PVI groups were 3.7 ± 1.0 and 3.3 ± 0.7 h, respectively (*p* = .07). The fluoroscopy times in the two groups were 20.8 ± 10.4 and 20.9 ± 8.2 min, respectively (*p* = .57; Table 2). There was no significant difference in sinus rhythm heart rates in those having ET‐GP ablation (median, 70; interquartile range [IQR], 15) and PVI (median, 67; IQR, 18) at 24‐h post ablation.

**Table 2:** Procedural details

Procedural details	PVI (*n* = 36)	GP ablation (*n* = 31)	*p* value
RF total energy used (kWs)	55.7 ± 22.7	23.3 ± 14.1	<.0001
Fluoroscopy time (min)	20.9 ± 8.2	20.8 ± 10.4	.57
Duration (h)	3.3 ± 0.7	3.7 ± 1.0	.07

*Note*: Values are presented as mean ± *SD* or *n* (%).

Abbreviations: GP, ganglionated plexus; PVI, pulmonary vein isolation; RF, radiofrequency.

There was one groin hematoma that was conservatively managed in the PVI group. In the ET‐GP ablation group, there was one patient with phrenic nerve palsy which was transient and fully resolved within 24 h. Our HFS mapping protocol did not include mapping within the PVs. However, when a GP was identified with HFS, we tried to delineate its boundaries and occasionally, this would cross the PV ostial border. Ablating in this region may have caused the transient phrenic nerve palsy in this patient.

### 3.2 Follow‐up

**Figure 4:**
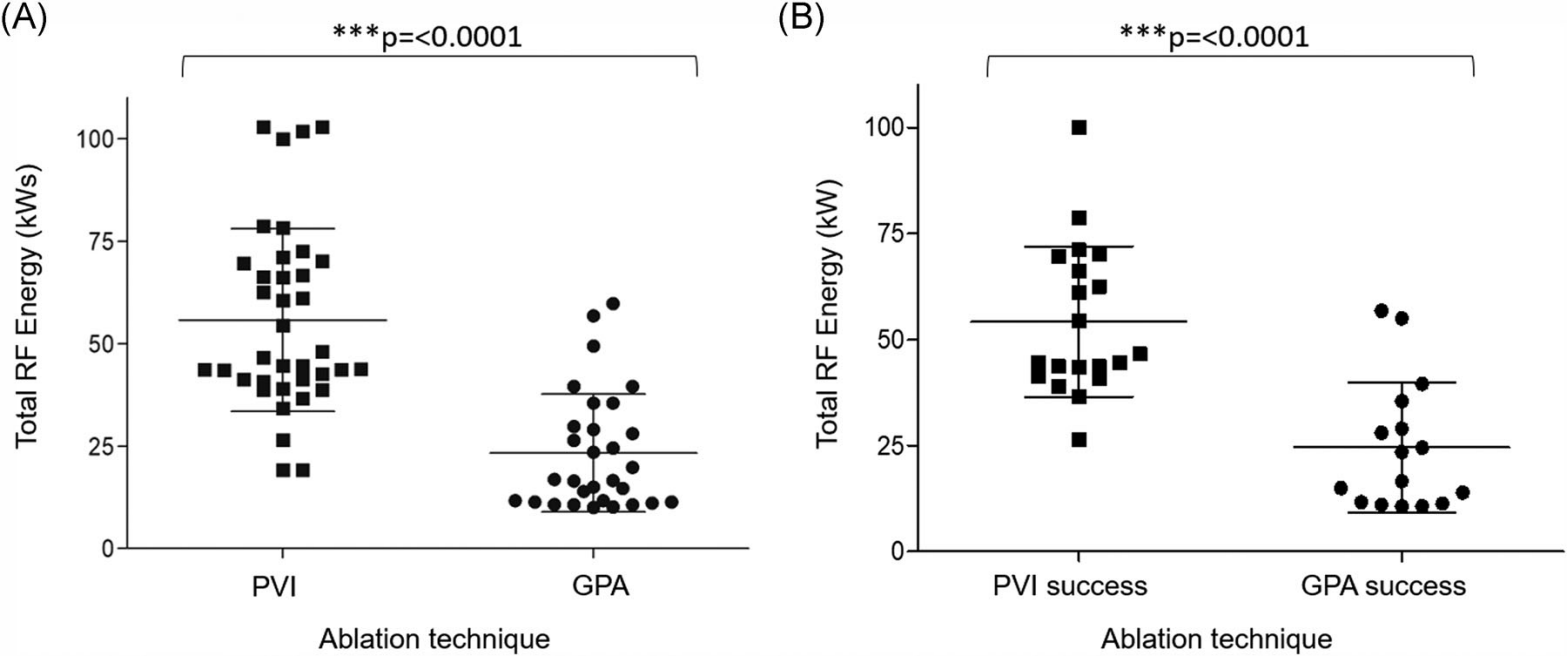
Average RF energy used in PVI and GP ablation. (A) A comparison of the average RF energy used between patients having PVI and GPA. The average RF energy used in PVI and GPA were 55.7 ± 22.3 and 23.3 ± 14.4 kWs, respectively (*p* ≤ .0001). (B) A comparison of the average RF energy used between patients having PVI and GPA who did not reach primary end‐point. The average RF energy used in successful PVI and GPA were 54.2 ± 17.7 and 24.6 ± 15.3 kWs, respectively (*p* ≤ .0001). GPA, ganglionated plexus ablation; PVI, pulmonary vein isolation; RF, radiofrequency

At 12‐month follow‐up with the ITT study population, 61% and 49% were free from ≥30 s of AF/AT or repeat ablation with PVI and ET‐GP ablation, respectively (log‐rank *p* = .27). Similarly, with the PP study population, 61% and 48% were free from ≥30 s of AF/AT or repeat ablation with PVI and ET‐GP ablation, respectively (log‐rank *p* = .28). The average RF energy used in the ET‐GP group was 23.3 ± 4.1 compared with 55.7 ± 22.7 kWs in the PVI group (*p* ≤ .0001; Figure 4A; for reference, 25 W applied for 20 min is a total of 30 kWs). Therefore, although there was no significant difference between PVI and ET‐GP ablation outcomes, PVI required far more ablation than ET‐GP ablation. Also, the average RF energy used in successful PVI was greater at 54.2 ± 17.7 kWs compared with 24.6 ± 15.3 kWs in successful ET‐GP ablations (*p* ≤ .0001; Figure 4B). There was no significant difference in mean heart rate between the two groups at 3, 6, 9, 12 months post ablation.

**Figure 5:**
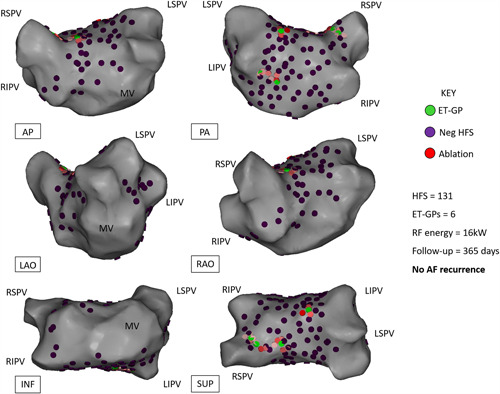
Example of a patient who had ET‐GP ablation and free from AF/AT at 12‐month follow‐up. Different projections of the left atrial CARTO™ 3D map are shown. Six ET‐GPs were identified and ablated. This was a 53‐year‐old male with hypertension, normal left ventricular systolic function, and normal left atrial size. AP, anterior–posterior; ET‐GP, ectopy‐triggering ganglionated plexus; HFS, high‐frequency stimulation; INF, inferior; LAO, left anterior oblique; LIPV, left inferior pulmonary vein; LSPV, left superior pulmonary vein; PA, posterior‐anterior; RAO, right anterior oblique; RF, radiofrequency; RIPV, right inferior pulmonary vein; RSPV, right superior pulmonary vein; SUP, superior

**Figure 6:**
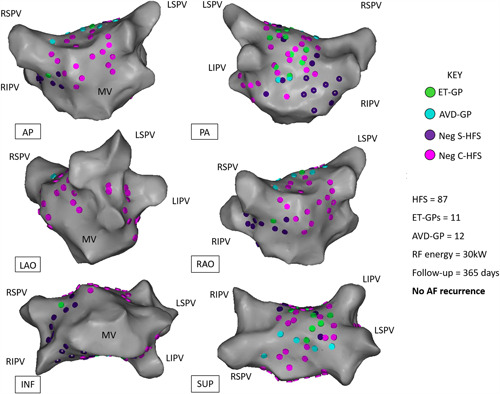
Example of a patient who had both ET‐GP and AVD‐GP ablated and free from AF/AT at 12‐month follow‐up. Different projections of the left atrial CARTO™ 3D map are shown. In total, 11 ET‐GPs and 12 AVD‐GPs were identified and ablated. This was a 63‐year‐old male with hypertension, diabetes mellitus, previous percutaneous coronary intervention to the right coronary artery, normal left ventricular systolic function, and mildly dilated left atrium. AP, anterior‐posterior; AVD‐GP, atrioventricular‐dissociating ganglionated plexus; C‐HFS, continuous high‐frequency stimulation; ET‐GP, ectopy‐triggering ganglionated plexus; HFS, high‐frequency stimulation; INF, inferior; LAO, left anterior oblique; LIPV, left inferior pulmonary vein; LSPV, left superior pulmonary vein; PA, posterior–anterior; RAO, right anterior oblique; RF, radiofrequency; RIPV, right inferior pulmonary vein; RSPV, right superior pulmonary vein; S‐HFS, synchronized high‐frequency stimulation; SUP, superior

**Figure 7:**
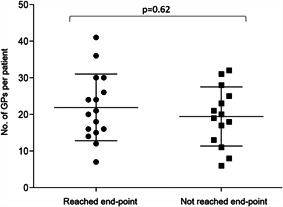
A scatter plot of the number of GPs (ET‐GP and AVD‐GP) identified in patients undergoing GP ablation. Sixteen patients who reached the primary end‐point had an average of 22 ± 9 GPs ablated. Fifteen patients who did not reach primary end‐point had an average of 19 ± 8 GPs ablated (*p* = .62). The longest line within the scatter plots represents the mean and the error bars represent standard deviation. AVD‐GP, atrioventricular dissociating ganglionated plexus; ET‐GP, ectopy‐triggering ganglionated plexus; GP, ganglionated plexus

Examples of a successful ET‐GP ablation is shown in Figure 5 and from a combined ET‐GP and AVD‐GP ablation in Figure 6. In the ET‐GP ablation group, 15 patients who did not reach primary endpoint had 19 ± 8 GPs, and 16 patients who reached primary end‐point had 21 ± 9 GPs (*p* = .62; Figure 7). Eight patients in the ET‐GP ablation group and five in the PVI group had repeat ablations for AF or AT within 365 days of their index procedure (Table S2).

## 4 DISCUSSION

This is the first study to functionally localize and ablate ET‐GP in the left atrium of patients with paroxysmal AF. We showed that it is safe and feasible to ablate ET‐GP, with an almost 50% efficacy at preventing AF. This was despite the learning curve we experienced during this study. ET‐GP ablation prevented AF using approximately 2.5 times less ablation energy than PVI (*p* ≤ .0001). There was no significant procedure time difference between the two groups, despite mapping on average 103 sites with HFS per patient. This was likely attributed to the shorter duration of ablation for ET‐GPs compared with PVI. There were a larger proportion of ET‐GPs identified per patient (20%) compared with AVD‐GPs which we previously mapped in a demographically similar cohort of patients (13%).^13^ Interestingly, there was no direct relationship between the number of ET‐GPs ablated and the prevention of AF.

### 4.1 Anatomical distribution of ET‐GP

Most ET‐GP were anatomically located around the PV antra, the roof, and down the midline of the anterior wall.^15^ Malcolme‐Lawes et al.^18^ previously demonstrated that PVI can abolish the PV ectopy triggering effect of ET‐GP in patients with AF. Other studies have also detected myocardium with unique fast Fourier‐transform characteristics in this region that has been associated with improved ablation outcomes.^19^ This may explain why some patients remain free from AF despite electrical reconnection of PVs. The ET‐GP located in the mid‐anterior wall and mid‐roof are not targeted by conventional PVI lines, which may explain the recurrence of AF despite complete PVI in some patients.

### 4.2 Approaches to autonomic modulation

Two studies have previously performed “selective” GP ablation in the human left atrium to map for AVD‐GP using continuous HFS.^20^, ^21^ The larger study of 80 patients limited functional testing to specific regions of the atria thought to contain GPs and tested 37 HFS sites spread across both atria, which yielded approximately five AVD‐GP per patient.^21^ This method of “selective” GP ablation performed significantly worse than “anatomical” GP ablation at preventing AF (42.5% vs. 77.5%; *p* = .02). However, two studies from the same group showed that “anatomical” GP ablation alone performed significantly worse at preventing AF than PVI.^22^ Addition of PVI to anatomical GP ablation produced more promising results, achieving significantly higher success at preventing AF than PVI alone^23^ (74% vs. 56%; *p* = .004). This was not a reproducible finding in the thoracoscopic GP ablation in addition to PVI for advanced AF (AFACT study)^24^; in fact, GP ablation in addition to PVI had significantly higher complication rates than PVI alone, including major bleeding, sinus node dysfunction, and pacemaker implantations. Four patients died after 1 year in the GP ablation group and none in the PVI group (*p* = .055). Continuous HFS was used to verify anatomical GP locations, though not all expected GP areas provoked an atrioventricular dissociating response which was ablated regardless.

We previously showed that both ET‐GP and AVD‐GP have a variable but discrete anatomical distribution in the left atrium which does not conform to all the anatomical areas that are known to contain GPs.^13^, ^15^, ^16^ ET‐GP identified with synchronized HFS produces PV and non‐PV ectopy that are reminiscent of clinical AF.^15^ However, synchronized HFS can only be performed in sinus rhythm, which may be challenging in patients who develop sustained AF during mapping, as this requires multiple DC cardioversions. It is well known that the precise location of GP vary between hearts significantly,^9^ as was the finding in this study. It is only by detailed global functional mapping with the use of appropriate HFS technique that we can identify all the relevant GP.

## 5 LIMITATIONS

The outcome data for GP ablation may be affected by the learning curve we experienced at the start of the study. We had a large number of patients crossed over from initial randomization to ET‐GP ablation to PVI due to sustained AF. Maintenance of sinus rhythm was essential to complete a thorough global map of ET‐GP. This was sometimes not possible despite multiple electrical cardioversions, and therefore, some ET‐GP may have been missed. We checked for reproducibility of atrial ectopy triggered by ET‐GP, but this does not completely reject the possibility of mechanical irritation from the catheter, and not a true autonomic stimulation effect. General anesthesia was used on all patients, and we do not know what impact this would have had on the threshold for triggering atrial ectopy and AF with GP stimulation. As the procedures were performed between 2013 and 2017, more modern ablation tools such as ablation index were not used during PVI or GP ablation. Recurrences of AF/AT were mainly documented from the three monthly 48‐h Holter ECGs. Asymptomatic arrhythmias outside the Holter monitoring period may have been missed during follow‐up.

## 6 CONCLUSION

It is feasible to perform detailed global functional mapping with HFS and ablate ET‐GP to prevent AF. This provides direct evidence that ET‐GP are part of the AF mechanism. Freedom from AF/AF with PVI and ET‐GP ablation was similar, but ET‐GP ablation required approximately 2.5 times less ablation than PVI. This indicates that GP ablation is a more specific target in the mechanism of AF. This proof‐of‐concept study provides a novel endpoint for AF ablation and justifies further investigation of the role of ET‐GP in AF pathophysiology.

## GROUP/CONSORTIUM MEMBERS

Ian Mann (Department of Cardiology, Hammersmith Hospital, Imperial College Healthcare NHS Trust, London, UK; Imperial Centre for Cardiac Engineering, Imperial College London, London, UK), Vishal Luther PhD (Department of Cardiology, Hammersmith Hospital, Imperial College Healthcare NHS Trust, London, UK; Imperial Centre for Cardiac Engineering, Imperial College London, London, UK), Kevin Leong PhD (Department of Cardiology, Hammersmith Hospital, Imperial College Healthcare NHS Trust, London, UK; Imperial Centre for Cardiac Engineering, Imperial College London, London, UK), Fu Siong Ng PhD (Myocardial Function Section, National Heart and Lung Institute, Imperial College London, London, UK;  Department of Cardiology, Hammersmith Hospital, Imperial College Healthcare NHS Trust, London, UK; Imperial Centre for Cardiac Engineering, Imperial College London, London, UK), Afzal Sohaib PhD (Department of Cardiology, Hammersmith Hospital, Imperial College Healthcare NHS Trust, London, UK; Imperial Centre for Cardiac Engineering, Imperial College London, London, UK), Michael Fudge (Department of Cardiology, Hammersmith Hospital, Imperial College Healthcare NHS Trust, London, UK; Imperial Centre for Cardiac Engineering, Imperial College London, London, UK), Elaine Lim (Department of Cardiology, Hammersmith Hospital, Imperial College Healthcare NHS Trust, London, UK; Imperial Centre for Cardiac Engineering, Imperial College London, London, UK), Michelle Todd (Department of Cardiology, Hammersmith Hospital, Imperial College Healthcare NHS Trust, London, UK; Imperial Centre for Cardiac Engineering, Imperial College London, London, UK), Ian Wright (Department of Cardiology, Hammersmith Hospital, Imperial College Healthcare NHS Trust, London, UK; Imperial Centre for Cardiac Engineering, Imperial College London, London, UK), Norman Qureshi PhD (Department of Cardiology, Hammersmith Hospital, Imperial College Healthcare NHS Trust, London, UK; Imperial Centre for Cardiac Engineering, Imperial College London, London, UK), Nicholas S. Peters MD (Myocardial Function Section, National Heart and Lung Institute, Imperial College London, London, UK;  Department of Cardiology, Hammersmith Hospital, Imperial College Healthcare NHS Trust, London, UK; Imperial Centre for Cardiac Engineering, Imperial College London, London, UK).

REFERENCES1

Calkins
H
, 
Hindricks
G
, 
Cappato
R
, et al. 2017 HRS/EHRA/ECAS/APHRS/SOLAECE expert consensus statement on catheter and surgical ablation of atrial fibrillation. Heart Rhythm. 2017;14:e275‐e444.2850691610.1016/j.hrthm.2017.05.012PMC60193272

Haïssaguerre
M
, 
Jaïs
P
, 
Shah
DC
, et al. Spontaneous initiation of atrial fibrillation by ectopic beats originating in the pulmonary veins. N Engl J Med. 1998;339:659‐666.972592310.1056/NEJM1998090333910033

Piccini
JP
, 
Lopes
RD
, 
Kong
MH
, 
Hasselblad
V
, 
Jackson
K
, 
Al‐Khatib
SM
. Pulmonary vein isolation for the maintenance of sinus rhythm in patients with atrial fibrillation a meta‐analysis of randomized, controlled trials. Circ Arrhythmia Electrophysiol. 2009;2:626‐633.10.1161/CIRCEP.109.856633200090774

Andrade
JG
, 
Champagne
J
, 
Dubuc
M
, et al. Cryoballoon or radiofrequency ablation for atrial fibrillation assessed by continuous monitoring. Circulation. 2019;140:1779‐1788.3163053810.1161/CIRCULATIONAHA.119.0426225

Kuck
KH
, 
Brugada
J
, 
Fürnkranz
A
, et al. Cryoballoon or radiofrequency ablation for paroxysmal atrial fibrillation. N Engl J Med. 2016;374:2235‐2245.2704296410.1056/NEJMoa16020146

Nery
PB
, 
Belliveau
D
, 
Nair
GM
, et al. Relationship between pulmonary vein reconnection and atrial fibrillation recurrence: a systematic review and meta‐analysis. JACC Clin Electrophysiol. 2016;2:474‐483.2975986810.1016/j.jacep.2016.02.0037

Nanthakumar
K
, 
Plumb
VJ
, 
Epstein
AE
, 
Veenhuyzen
GD
, 
Link
D
, 
Kay
GN
. Resumption of electrical conduction in previously isolated pulmonary veins. Circulation. 2004;109:1226‐1229.1499312410.1161/01.CIR.0000121423.78120.498

Li
S
, 
Scherlag
BJ
, 
Yu
L
, et al. Low‐level vagosympathetic stimulation. Circ Arrhythmia Electrophysiol. 2009;2:645‐651.10.1161/CIRCEP.109.868331199485059

Ardell
JL
, 
Armour
JA
. Neurocardiology: structure‐based function. Compr Physiol. 2016;6:1635‐1653.2778385410.1002/cphy.c15004610

Hou
Y
, 
Scherlag
BJ
, 
Lin
J
, et al. Interactive atrial neural network: determining the connections between ganglionated plexi. Heart Rhythm. 2007;4:56‐63.1719899110.1016/j.hrthm.2006.09.02011

Schauerte
P
, 
Scherlag
BJ
, 
Patterson
E
, et al. Focal atrial fibrillation: experimental evidence for a pathophysiologic role of the autonomic nervous system. J Cardiovasc Electrophysiol. 2001;12:592‐599.1138652210.1046/j.1540-8167.2001.00592.x12

Lemery
R
, 
Birnie
D
, 
Tang
ASL
, 
Green
M
, 
Gollob
M.

Feasibility study of endocardial mapping of ganglionated plexuses during catheter ablation of atrial fibrillation. Heart Rhythm. 2006;3:387‐389.1656728310.1016/j.hrthm.2006.01.00913

Kim
MY
, 
Sikkel
MB
, 
Hunter
RJ
, et al. A novel approach to mapping the atrial ganglionated plexus network by generating a distribution probability atlas. J Cardiovasc Electrophysiol. 2018;29:1624‐1634.3016823210.1111/jce.13723PMC636968414

Lim
PB
, 
Malcolme‐Lawes
LC
, 
Stuber
T
, et al. Stimulation of the intrinsic cardiac autonomic nervous system results in a gradient of fibrillatory cycle length shortening across the atria during atrial fibrillation in humans. J Cardiovasc Electrophysiol. 2011;22:1224‐1231.2161581410.1111/j.1540-8167.2011.02097.x15

Kim
MY
, 
Sandler
B
, 
Sikkel
MB
, et al. The ectopy‐triggering ganglionated plexuses in atrial fibrillation. Auton Neurosci. 2020;228:102699.3276902110.1016/j.autneu.2020.102699PMC751159916

Kim
MY
, 
Sandler
BC
, 
Sikkel
MB
, et al. Anatomical distribution of ectopy‐triggering plexuses in patients with atrial fibrillation. Circ Arrhythmia Electrophysiol. 2020;44:1045‐1047.10.1161/CIRCEP.120.0087153271818717

Lim
PB
, 
Malcolme‐Lawes
LC
, 
Stuber
T
, et al. Intrinsic cardiac autonomic stimulation induces pulmonary vein ectopy and triggers atrial fibrillation in humans. J Cardiovasc Electrophysiol. 2011;22:638‐646.2123567110.1111/j.1540-8167.2010.01992.x18

Malcolme‐Lawes
LC
, 
Lim
PB
, 
Wright
I
, et al. Characterization of the left atrial neural network and its impact on autonomic modification procedures. Circ Arrhythmia Electrophysiol. 2013;6:632‐640.10.1161/CIRCEP.113.0001932358074319

Pachon
MJC
, 
Pachon
MEI
, 
Pachon
MJC
, et al. A new treatment for atrial fibrillation based on spectral analysis to guide the catheter RF‐ablation. Europace. 2004;6:590‐601.1551926310.1016/j.eupc.2004.08.00520

Scanavacca
M
, 
Pisani
CF
, 
Hachul
D
, et al. Selective atrial vagal denervation guided by evoked vagal reflex to treat patients with paroxysmal atrial fibrillation. Circulation. 2006;114:876‐885.1692375710.1161/CIRCULATIONAHA.106.63356021

Pokushalov
E
, 
Romanov
A
, 
Shugayev
P
, et al. Selective ganglionated plexi ablation for paroxysmal atrial fibrillation. Heart Rhythm. 2009;6:1257‐1264.1965673610.1016/j.hrthm.2009.05.01822

Katritsis
D
, 
Giazitzoglou
E
, 
Sougiannis
D
, 
Goumas
N
, 
Paxinos
G
, 
Camm
AJ
. Anatomic approach for ganglionic plexi ablation in patients with paroxysmal atrial fibrillation. Am J Cardiol. 2008;102:330‐334.1863859610.1016/j.amjcard.2008.03.06223

Katritsis
DG
, 
Pokushalov
E
, 
Romanov
A
, et al. Autonomic denervation added to pulmonary vein isolation for paroxysmal atrial fibrillation: a randomized clinical trial. J Am Coll Cardiol. 2013;62:2318‐2325.2397369410.1016/j.jacc.2013.06.05324

Driessen
AHG
, 
Berger
WR
, 
Krul
SPJ
, et al. Ganglion plexus ablation in advanced atrial fibrillation. J Am Coll Cardiol. 2016;68:1155‐1165.2760967610.1016/j.jacc.2016.06.036

## Supporting information

Supporting information.

## Data Availability

The data that support the findings of this study are available from the corresponding author upon reasonable request.
