# Supplementary material for: Targeting the ectopy‐triggering ganglionated plexuses without pulmonary vein isolation prevents atrial fibrillation
Source: J Cardiovasc Electrophysiol. 2021 Jan 19;32(2):235–44. doi: 10.1111/jce.14870 (PMC8611799; doi:10.1111/jce.14870)
Supplement: Supplementary file 1 — Supporting information. [file JCE-32-235-s001.docx]

**SUPPLEMENTARY MATERIALS**

**Supplementary Table 1. Inclusion and exclusion criteria of the study**

| **Inclusion criteria** | **Exclusion criteria** |
| --- | --- |
| - Males or females from 18 to 85yrs old - Paroxysmal atrial fibrillation - Off amiodarone for at least 60 days - Suitable candidate for catheter ablation - Signed informed consent | - Contraindication to catheter ablation - Contraindication for general anaesthetic - Presence of a left ventricular thrombus - Previous left atrial ablation - Valvular disease that is grade moderate or greater - Any form of cardiomyopathy - Severe cerebrovascular disease - Active gastrointestinal bleeding - Serum Creatinine >200umol/L or on dialysis or at risk of requiring dialysis - Active infection or fever - Life expectancy shorter than the duration of the trial - Allergy to contrast - Moderate to severe heart failure and/or NYHA Class III-IV - Bleeding or clotting disorders or inability to receive heparin - Uncontrolled diabetes (HbA1c ≥73mmol/mol or HbA1c ≤64mmol/mol and Fasting Blood Glucose ≥9.2mmol/L) - Malignancy needing therapy - Pregnancy or women of childbearing potential not using a highly effective method of contraception - Unable to give informed consent or has insufficient comprehension |

(NYHA=New York Heart Association)

**Supplementary Table 2. Repeat ablations after PVI and GP ablation.**

| **Further ablations up to 12 months post index procedure** | **Total**  **(n=67)** | **PVI**  **(n=36)** | **GPA**  **(n=31)** | **p Value** |
| --- | --- | --- | --- | --- |
| Total ablations | 13 (19%) | 5 (14%) | 8 (26%) | 0.28 |
| PVI | 9 (13%) | 3 (8%) | 6 (19%) | 1.0 |
| AT | 2 (3%) | 1 (3%) | 1 (3%) | 1.0 |
| PVI+AT | 1 (1%) | 1 (3%) | 0 (0) | 1.0 |
| AFL | 1 (1%) | 0 (0) | 1 (3%) | 1.0 |

Values are in n (%).

(AFL=atrial flutter, AT=atrial tachycardia, GPA=ganglionated plexus ablation, PVI=pulmonary vein isolation)
